# H3Africa partnerships to empower clinical research sites to generate high-quality biological samples

**DOI:** 10.4102/ajlm.v9i1.935

**Published:** 2020-03-18

**Authors:** Talishiea Croxton, Ndidi Agala, Emmanuel Jonathan, Olasinbo Balogun, Petronilla J. Ozumba, Enzenwa Onyemata, Shefiya Lawal, Manmak Mamven, Samuel Ajayi, Sylvia E. Melikam, Mayowa Owolabi, Bruce Ovbiagele, Dwomoa Adu, Akinlolu Ojo, Christine M. Beiswanger, Alash’le Abimiku

**Affiliations:** 1 Institute of Human Virology Nigeria, Abuja, Nigeria; 2 Institute of Human Virology, University of Maryland, Baltimore, Maryland, United States; 3 Department of Internal Medicine, University of Abuja Teaching Hospital, Abuja, Nigeria; 4 Department of Medicine, University of Abuja Teaching Hospital, Abuja, Nigeria; 5 Department of Medicine, University of Ibadan, Ibadan, Nigeria; 6 Center for Genomic and Precision Medicine, College of Medicine, University of Ibadan, Ibadan, Nigeria; 7 Department of Neurology, University of Ghana Medical School, Accra, Ghana; 8 Department of Neurology, Medical University of South Carolina, Charleston, South Carolina, United States; 9 Department of Internal Medicine, University of Michigan, Ann Arbor, Michigan, United States; 10 Coriell Institute for Medical Research, Camden, New Jersey; 11 Independent Contractor, Philadelphia, Pennsylvania, United States

**Keywords:** biobank, training, Africa, developing country, biotechnology

## Abstract

**Background:**

The Institute of Human Virology Nigeria (IHVN) – Human Heredity and Health in Africa (H3Africa) Biorepository (I-HAB) seeks to provide high-quality biospecimens for research. This depends on the ability of clinical research sites (CRS) – who provide biospecimens – to operate according to well-established industry standards. Yet, standards are often neglected at CRSs located in Africa. Here, I-HAB reports on its four-pronged approach to empower CRSs to prepare high-quality biospecimens for research.

**Objectives:**

I-HAB sought (1) to assess a four-pronged approach to improve biobanking practices and sample quality among CRSs, and (2) to build human capacity.

**Methods:**

I-HAB partnered with two H3Africa principal investigators located in Nigeria and Ghana from August 2013 through to May 2017 to debut its four-pronged approach (needs assessment, training and mentorship, pilot, and continuous quality improvement) to empower CRSs to attain high-quality biospecimens.

**Results:**

Close collaborations were instrumental in establishing mutually beneficial and lasting relationships. Improvements during the 12 months of engagement with CRSs involved personnel, procedural, and supply upgrades. In total, 51 staff were trained in over 20 topics. During the pilot, CRSs extracted 50 DNA biospecimens from whole blood and performed quality control. The CRSs shipped extracted DNA to I-HAB and I-HAB that comparatively analysed the DNA. Remediation was achieved via recommendations, training, and mentorship. Preanalytical, analytical and post-analytical processes, standard operating procedures, and workflows were systematically developed.

**Conclusion:**

Partnerships between I-HAB and H3Africa CRSs enabled research sites to produce high-quality biospecimens through needs assessment, training and mentorship, pilot, and continuous monitoring and improvement.

## Introduction

Biobanking is underdeveloped in Africa.^1,2^ Obvious obstacles include sparse financial resources, challenging operating environments, underdeveloped infrastructure, inferior logistics, and an unreliable electrical power supply.^2,3,4,5^ Also, preanalytical processes occurring at collection sites affect biospecimen quality.^3^ Past reports documented destitute laboratory systems, and the hope for advancements through international declarations, funding, networking, training, and mentorship.^6,7,8,9^

The United States National Institutes of Health (NIH) and the Wellcome Trust founded Human Heredity and Health in Africa (H3Africa) (www.h3Africa.org) to promote genomic research in Africa through funding of African researchers, and provision of bioinformatics core and biorepositories located in Nigeria, South Africa, and Uganda.^6^ The NIH required biorepositories to complete a feasibility phase (Phase I) to qualify for the implementation phase (Phase II). The NIH also assigned H3Africa research projects to a biorepository and required projects to deposit an aliquot of all DNA. The bioinformatics core stored data. Beiswanger et al.^10^ described the process for accessing biospecimens.

The NIH funded the Institute of Human Virology Nigeria H3Africa Biorepository (I-HAB). I-HAB’s primary pursuit in Phase I was to achieve international standards according to the International Society for Biological and Environmental Repositories (ISBER) best practices.^11^ For example, ISBER provides requirements for biospecimen collection, processing, quality control (QC), storage and transport, infrastructure, quality management, safety, training, and a laboratory information management system (LIMS).^11^ The LIMS documents and tracks biospecimen attributes from collection through to use, depletion, and destruction. I-HAB also referred to the International Organization for Standardization (ISO) 15189 standards for clinical laboratories (https://www.iso.org/standard/56115.html) and Strengthening Laboratory Management Toward Accreditation (SLMTA) (https://slmta.org) for requirements pertaining to quality management, as appropriate. Regarding specific protocols, I-HAB referred to manufacturer specifications and journals.

Considering the potential imbalance between standardised preanalytical processes according to ISBER, ISO, and SLMTA and the lack of such standards in laboratories in Africa, I-HAB sought to evaluate assigned clinical research sites (CRSs) and implement improvement strategies. I-HAB established a training and mentorship programme to bridge gaps and to ensure researchers obtain biospecimens of the highest quality. This article describes I-HAB’s experience in engaging two H3Africa projects from August 2013 through May 2017 in preparation for Phase II.^10^

## Methods

The NIH assigned I-HAB six research projects including Project A – a CRS located in Tanzania (not included in this study), Nigeria, and Ghana, with the project’s central laboratory hub (clinical research sites sends biospecimens to laboratory hubs for processing, testing or storage) in Ghana – and Project B, with a CRS and laboratory hub in Nigeria. I-HAB employed a four-pronged approach of needs assessment, training and mentorship, piloting, and continuous quality improvement to help sites to collect, process, store, and transport high-quality biospecimens for future research.

A working group of NIH experts and representatives from each H3Africa biorepository developed guidelines and protocols to guide the H3Africa consortium on biorepository processes for harmonisation and consistency. The documents included biospecimens deposit and access requirements, and specific procedures for biospecimen collection, processing, transport, and shipping (www.h3africa.org). For example, the group established minimal criteria for DNA purity and concentration to ensure quality and consistency. The guidelines required submitters to maintain:

- **Ethical standards:** ethical approval, informed consent, and Material Transfer Agreements (MTA).
- **Legal requirements:** shipping regulations and import and export permits.
- **H3Africa requirements:** DNA quality, minimal data set, shipping checklist, and manifest and query forms. The ‘minimal data set’ included a biospecimen identification code, de-identified participant identification code, study name, specimen type, date of collection, gender, age at collection, and storage box position. The working group standardised the format of each element to ensure consistency and decrease errors.

The working group based the contents of the standard operating procedures (SOPs) on International Air and Transport Association regulations, ISBER best practices, manufacturer’s specifications, and well-established practices as specified. ISO 15189 standards were useful for creating SOPs and documents regarding quality management and for defining the structural elements required for all SOPs. I-HAB recommended that sites customise SOPs to their laboratories.

### Ethical Considerations

This article followed all ethical standards for research without direct contact with human or animal subjects.

### Needs assessment

The I-HAB and research teams met to discuss project descriptions, processes, and procedures that potentially impact a biospecimen’s quality. I-HAB reviewed study protocols and other documents in consideration of responsibilities and procedures impacting a biospecimen’s integrity. I-HAB assessed facilities, equipment, personnel, documents and records, specimen management, organisation, purchasing, inventory, and quality assurance measures in line with underlying principles of ISBER best practices and ISO 15189 to ensure the sites could meet protocol requirements according to industry standards. Assessments included a combination of discussion, SOP review, site observation, and competency assessments for specifically assigned tasks. I-HAB offered document development, training and mentorship, biospecimen storage, distribution, and QC to resolve problems identified by assessments.

### Training and mentorship

I-HAB customised training and mentorship for Project A’s clinical site in Nigeria and its hub, and Project B’s hub site according to the outcome of the needs assessment. Training and mentorship occurred at I-HAB, CRSs and hubs for groups and individuals. Training began with lectures to introduce theories, followed by exercises to provide practical experience, and concluded with participant assessments to test competency. Mentorship provided additional time to practise procedures, to address problems revealed through training, and to review processes that were required but were not covered during training. Mentorship areas included document development and revision, workflow design, supply chain management, biological transportation, and laboratory procedures.

### Pilot study

Following training and mentorship, I-HAB piloted exercises with Project A and Project B to determine preparedness for biospecimen deposition. Sites tested processes preceding biospecimen deposit including participant recruitment, biospecimen collection, processing, QC, temporary storage, transport to I-HAB, and documentation. I-HAB tested biorepository processes succeeding biospecimens deposit including: biospecimen receipt, QC, aliquoting, storage, transport, and documentation. I-HAB and the sites processed MTAs, and I-HAB acquired copies of ethical approvals and blank consent forms to ensure biospecimen sharing was according to ethical requirements. Investigators transferred biospecimens collected from geographically dispersed CRSs to the study’s hub site and the hub site transferred DNA biospecimens to I-HAB for comparative QC and storage. Project A’s CRS in Nigeria was the only one to transfer biospecimens directly to I-HAB, because it required assistance to temporarily store and ship non-DNA biospecimens to its hub in Ghana. Thus, I-HAB piloted shipments from Project A’s and Project B’s hubs to I-HAB, from Project A’s site in Nigeria to I-HAB and from I-HAB to Project A’s hub.

#### Shipping

Sites shipped biospecimens according to International Air and Transport Association regulations under Class 6.2 biologicals, infectious substances, and Class 9 for dry ice (unless otherwise stated). Shipments contained Temp Tale4 quantitative, reusable temperature loggers, (Sensitech, Beverly, Massachusetts, United States) and 3TM Warmmark Time Temperature Indicators (qualitative, disposable cards) (TelaTemp, Anaheim, California, United States) unless otherwise indicated. Biospecimens were documented upon receipt.

#### Quality control

I-HAB required sites to submit QC results for the DNA deposited. I-HAB also evaluated DNA for concentration (absorbance at 260 nanomole [nm]) and purity (260/280 absorbance ratio) by spectrophotometry (NanoDrop, ThermoScientific, Waltham, Massachusetts, United States), and for quality by gel electrophoresis.^12,13^ DNA purity of 1.7–2.0 was acceptable. DNA with unacceptable purity was stored but flagged for suboptimal quality. Where specified, I-HAB and Project A’s Nigerian site investigated plasma and serum for haemolysis using I-HAB’s protocol for visual grading. Laboratorians classified biospecimens from 0 (normal) to 4 (extreme haemolysis) by comparing them to a picture gradient of five adjacent plasma samples (numbered 0 to 4), beginning with a normal, non-haemolysed sample and gradually increasing to an extremely haemolysed sample.

I-HAB documented and communicated outcomes and recommendations to the projects’ principal investigators and staff.

### Continuous quality improvement

I-HAB engaged sites in continuous quality improvement. Continuous improvement is a cyclical process to improve quality by identifying problems and resolving them. I-HAB reviewed biospecimen QC, and shipment packaging, temperature, and documentation. I-HAB provided additional training or pilot exercises where appropriate to address nonconformities. The teams implemented corrective and preventive measures before initiating Phase II shipments to I-HAB. I-HAB determined improvement by the elimination of previously identified problems, improvements in DNA quality, and overall improvements in efficiency.

Following pilot deposition at I-HAB, Project A and Project B continued to identify opportunities for improvement. Project hubs monitored temperatures in shipments between I-HAB and project hubs. I-HAB monitored biospecimens and corresponding data for all subsequent deposits and communicated discrepancies to the sender within one week of discovery. Similarly, hubs continued to QC 100% of the DNA deposited. To ensure accuracy, I-HAB re-tested 10% of the DNA received. If more than 10% of the re-tested DNA was beyond acceptable range or inconsistent with I-HAB’s results, then I-HAB tested the remaining 90% (total 100%). In such instances, I-HAB tested 100% of subsequent batch shipments from the project, until the project completed two consecutive shipments for which the QC for at least 90% of the DNA tested was within acceptable range and consistent with I-HAB’s results. I-HAB continued to meet with the projects’ staff to discuss future activities, challenges, and strategies of improvement.

## Results

Engagement activities occurred over the three years of Phase I (2013–2016), in preparation for Phase II. I-HAB observed improvements in staff proficiency, procedural efficiency, and supply upgrades. I-HAB trained 51 persons in over 20 topics. I-HAB further determined improvements in supplies by the elimination of previously identified issues that resulted in poor sample quality. Preanalytical, analytical, and post-analytical processes, documents, and workflows were systematically created and modified based on needs assessment, training, mentorship, pilot exercises, and continuous quality improvement.

### Project A

**FIGURE 1:**
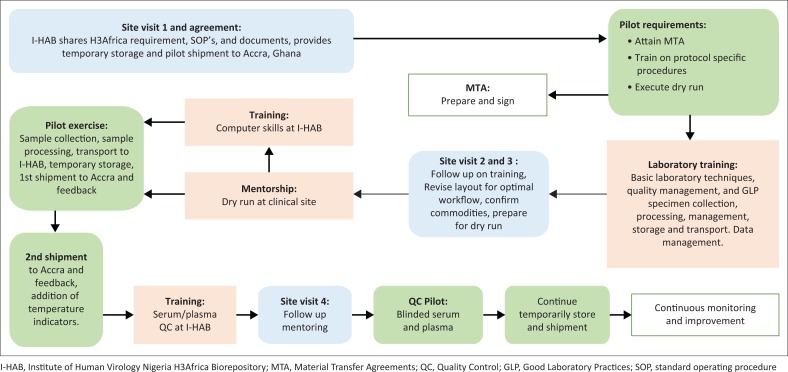
Partnership to empower Project A’s clinical sites in Nigeria and Ghana. I-HAB engaged clinical sites through needs assessment, training and mentorship, pilot exercise, and continuous monitoring in preparation for sample deposition.

The study’s investigators established a system for Phase II. The CRS in Nigeria collected, processed, temporarily stored, and shipped biospecimens to the project’s hub in Ghana. An independent, commercial contractor extracted and QC tested DNA. The hub stored DNA and shipped the aliquots required for deposition to I-HAB. I-HAB performed needs assessment, training, and the pilot of the entire study process with the site in Nigeria, then piloted DNA shipment from Ghana to I-HAB. The process of engagement with Project A’s CRS in Nigeria is summarised in Figure 1.

#### Needs assessment

In March 2014, I-HAB’s manager, supervisor and QC officer assessed the CRS in Nigeria to compare infrastructural, commodity, and staffing resources to the laboratory protocol, H3Africa requirements, and best practices according to ISBER. The staff included an entry-level laboratory technician and data clerk. I-HAB evaluated the laboratory, staff, equipment, supplies, procedures, and workflows. The I-HAB manager communicated assessment outcomes and recommendations to the site principal investigator, study coordinator, laboratory technician, and data clerk as appropriate. I-HAB and Project A agreed that training, mentorship, and document development were required for Phase II preparation.

#### Training and mentorship

I-HAB provided training and mentorship for Project A’s clinical site laboratory technician and data clerk:

- The laboratory technician attended a five-day workshop at I-HAB, September 2013 (Table 1).
- I-HAB provided customised five-day training for the laboratory technician, incorporating her responsibilities per the study protocol and H3Africa specifications, January 2014 (Table 1).
- The I-HAB manager, supervisor, and biospecimens QC officer mentored the laboratory technician and data clerk for one day at their facility during a dry run on February 2014.
  ■ The dry run simulated activities from participant arrival through to mock biospecimen collection, processing, temporary storage, and transport, including documentation.
  ■ The laboratory technician packed and transported the mock biospecimens and the manifest using I-HAB’s daily shuttle from Project A to I-HAB. (The drivers were trained in biological safety and biospecimens transport.)
  ■ I-HAB verified all mock biospecimens against the manifest.
  ■ I-HAB assisted in the development or modification of worksheets as appropriate.
  ■ The dry run revealed that the lab technician was not proficient in computer operations.
- The technician participated in a four-day introductory computer workshop, March 2014.

**TABLE 1:** Training topics to build knowledge and skills – Project A in Nigeria.

Attendee type (No.)	Total number of staff trained	Venue	Training type (date)	Topics covered
Laboratory technician (1)	1	I-HAB	Laboratory orientation (September 2013)	Quality management systems
				Safety
				Good laboratory practices
				SOP writing
				Documentation
				Inventory management
				Laboratory organisation
				Use and care of basic lab equipment
				Overview of HIV infection and testing according to national algorithm
			Customised training (January 2014)	Biospecimen collection
				Biospecimen processing
				Biospecimen storage
				Organise and document biospecimen storage
				Biospecimen acceptance or rejection
				Local specimen transport
				QC: Plasma and serum visual grading
				Temperature monitoring
				Standard preanalytical code
				Minimum information about biobank data sharing (MIABIS)
				Intro to computers: General operations, Microsoft Word and Excel
Data clerk (4) Laboratory staff (5) Nurse (2) Research coordinator (1)	12	I-HAB	Customised training (March 2015)	Good laboratory practices
				Pipetting techniques
				Biospecimen collection
				Biospecimen labelling and aliquoting
				Biospecimen reception and rejection
				Plasma and serum processing
				QC: Plasma and serum visual grading
				DNA extraction: Theory and SOP only (no practical)
				QC: DNA
				Biospecimen storage
				Biospecimen shipment
				IATA regulations
				Standard preanalytical code MIABIS†
				Documents, records and data management
				Temperature monitoring
				Workflow

I-HAB, Institute of Human Virology Nigeria H3Africa Biorepository; QC, quality control; SOP, standard operating procedures; IATA, International Air and Transport Association; MIABIS, Minimum information about biobank data sharing.

† , A means to standardise data by defining minimal data elements that are common among biorepositories, such as data that describes samples.

During the two-day dry run, improvements maximised client and staff safety, specimen integrity, and efficiency. I-HAB rearranged the biospecimen collection and processing area to reflect the natural flow of activities. Samples were inadequately separated during processing, and storage vials popped once they were frozen. Thus, I-HAB made recommendations that improved biospecimen integrity: (1) upgrade the centrifuge to enable staff to process biospecimens with adequate centrifugal force; (2) separate and freeze plasma, red cells, and buffy coat immediately, rather than freeze whole blood in Ethylenediaminetetraacetic acid vacutainers without processing them, and (3) replace flip cap tubes with cryovials for biospecimen storage. I-HAB also provided Project A with order information for biological shipping materials.

#### Pilot

I-HAB performed two pilots with Project A. The first pilot tested the CRS’s processes of enrolment, samples processing, temporary storage, and shipment from I-HAB to Ghana. The second pilot contained one aliquot of all Project A’s DNA. The pilot tested the quality of the DNA and shipment from Ghana to Abuja. DNA was extracted at a commercial laboratory, stored at the hub and shipped to I-HAB on dry ice by DHL Nigeria (Lagos, Nigeria). I-HAB provided the hub with temperature loggers and indicators to monitor the shipment. Upon receipt, I-HAB investigated DNA quality using NanoDrop (ThermoScientific, Waltham, Massachusetts, United States) and agarose gel electrophoresis. Outcomes were discussed with project personnel and recommendations made.

#### Project A, clinical site (Nigeria)

The clinical research site pilot occurred in February 2014. The MTA took seven months to process, due to unfamiliarity with such agreements; subsequent MTAs took one to three months. The site collected and processed biospecimens, performed visual grading for haemolysis for plasma and serum, and documented the results in the manifest. Upon delivery, I-HAB inspected all biospecimens and imported the data into Freezerworks (Dataworks Development, Mountlake Terrace, Washington, United States). I-HAB’s QC officer blindly re-evaluated 40 of the plasma and serum using the same visual grading methods for comparison. 92.5% of the results were consistent. Also, I-HAB temporarily stored and subsequently shipped on dry ice 1075 biospecimens to the hub in Ghana using DHL Nigeria (Lagos, Nigeria): plasma, red cells, buffy coat, urine, and oral fluid. The shipment duration was two days and temperature monitors confirmed that targeted temperature ranges were maintained. I-HAB received favourable feedback regarding the shipment.

**FIGURE 2:**
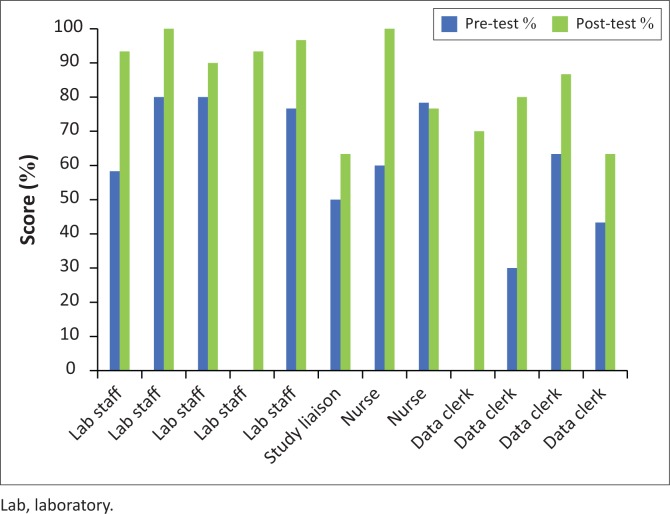
Project A’s training scores in Nigeria March 2015. Project A’s pre-test and post-test training scores according to staff designation. Two persons were absent during the pre-test; thus, those scores are missing.

Project A amended practices at its other CRSs to reflect improvements made during the pilot. In March 2015, I-HAB trained 12 persons from five Project A CRSs for one week at I-HAB (Table 1). Trainees were five laboratorians, one coordinator, two nurses, and four data clerks. Nine (90%) trainees who took pre-tests and post-tests increased in performance (Figure 2). The average pre-test scores were 62% (range 30% to 80%) while average post-test scores were 84.4% (range 63% to 100%). As expected, laboratorians performed better than non-laboratorians on average.

#### Project A, hub site (Ghana)

During Project A’s hub’s pilot in September 2015, Project A shipped 20 unique DNA biospecimens (extraction and QC at a commercial laboratory) to I-HAB by DHL on dry ice in four days. Ghana transferred data via a template comma-separated values file. The DNA was still frozen upon receipt, but temperature monitors were not included in the shipment. Also, there were inconsistencies between site versus biorepository results. Only 5% of the DNA was within the acceptable purity range per Ghana’s result versus 25% per I-HAB (Table 2). The average purity reported by both was below the acceptable standard. Furthermore, DNA concentration averaging 2.0 ng/*µ*l (0.1–7.1) was lower than the recommended for deposition by H3Africa consortium. NanoDrop 2000 and 8000 series have lower detection limits of 2 ng/*µ*l and 2.5 ng/*µ*l; thus, measurements below these limits are unreliable. The DNA was not evaluated by agarose gel electrophoresis due to poor DNA integrity. These samples were tagged as low quality before storage and results were communicated and discussed with Project A. The major benefits of the pilot were knowledge of Project A’s contractor’s poor DNA quality and non-compliance with the shipping procedures.

**TABLE 2:** DNA quality control results for Projects A and B.

Sample no.	Project A: Ghana†								Project B: Nigeria‡							
	First pilot shipment September 2015				First Phase II shipment April 2017				Pilot shipment June 2015				First Phase II shipment November 2016			
	Concentration (ng/*μ*1)		Purity (260/280)		Concentration (ng/*μ*1)		Purity (260/280)		Concentration (ng/*μ*1)		Purity (260/280)		Concentration (ng/*μ*1)		Purity (260/280)	
	I-HAB	Project A	I-HAB	Project A	I-HAB	Project A	I-HAB	Project A	I-HAB	Project B	I-HAB	Project B	I-HAB	Project B	I-HAB	Project B
1	3.10	1.30	1.79	**0.00**	187.90	161.70	**1.30**	**1.30**	204.60	115.00	1.86	1.89	49.34	63.50	1.87	1.82
2	7.10	1.90	2.10	**0.00**	198.90	197.50	**1.24**	**1.22**	71.50	74.90	1.79	1.83	52.25	58	1.83	1.85
3	2.30	0.00	1.41	**0.00**	134.60	138.30	**1.14**	**1.13**	16.80	17.50	1.78	1.73	78.47	77.80	1.87	1.87
4	2.00	0.10	2.09	**0.00**	127.00	143.20	1.86	**1.61**	53.30	55.00	1.81	1.91	49.34	54.70	1.84	1.81
5	1.80	0.50	2.29	**0.00**	160.90	275.90	**1.16**	**0.74**	48.00	46.00	1.78	1.88	76.87	95.00	1.86	1.85
6	0.10	0.00	0.26	**0.88**	213.40	223.30	**1.38**	**1.38**	**120.70**	**115.00**	**1.86**	**1.89**	136.50	147.10	1.84	1.86
7	6.30	3.30	1.79	**3.66**	154.50	143.70	**1.66**	**1.68**	**69.10**	**74.90**	**1.80**	**1.83**	93.86	99.40	1.85	1.84
8	0.20	3.20	1.71	**9.18**	300.30	345.00	1.71	1.88	**15.00**	**17.50**	**1.88**	**1.73**	190.60	175.60	1.83	1.78
9	0.20	0.00	2.48	**1.47**	174.10	247.00	1.41	**1.39**	**50.90**	**55.00**	**1.82**	**1.91**	162.80	159.90	1.80	1.81
10	2.30	2.90	1.68	1.97	152.00	63.00	1.49	**1.59**	**46.70**	**46.00**	**1.80**	**1.88**	80.11	81.5	1.84	1.95
11	1.00	0.20	2.27	0.00	336.00	320.80	1.69	1.72	-	-	-	-	61.07	66.20	1.84	1.88
12	1.40	0.00	1.10	0.86	152.80	139.90	1.11	**1.07**	-	-	-	-	83.82	86.40	1.82	1.77
13	1.90	1.30	1.31	4.70	149.40	164.70	1.40	**1.41**	-	-	-	-	74.11	66.70	1.81	1.79
14	3.90	0.00	1.77	1.22	136.40	126.80	1.71	1.75	-	-	-	-	87.65	95.10	1.86	1.86
15	0.10	0.00	0.14	0.85	259.60	242.40	1.73	1.84	-	-	-	-	132.5	130.6	1.82	1.87
16	0.50	0.00	1.19	0.90	172.20	201.10	**1.31**	**1.30**	-	-	-	-	83.41	92.20	1.86	1.84
17	0.80	0.00	1.69	1.21	232.00	216.30	**1.19**	**1.20**	-	-	-	-	62.40	65.10	1.86	1.80
18	1.00	3.10	1.21	2.63	178.30	247.60	**1.15**	**1.17**	-	-	-	-	87.18	81.30	1.89	1.84
19	3.00	0.40	1.69	0.00	78.72	Not Provided	**1.46**	**1.49**	-	-	-	-	61.51	78.10	1.75	1.79
20	1.30	0.00	1.87	0.12	143.70	198.60	**1.42**	**1.45**	-	-	-	-	90.71	98.20	1.83	1.77

**Average**	**2.02**	**0.91**	**1.59**	**1.48**	**209.78**	**222.50**	**1.54**	**1.57**	**69.66**	**61.68**	**1.82**	**1.85**	**89.73**	**93.62**	**1.84**	**1.83**
**Minimum**	**0.10**	**0.00**	**0.14**	**0.00**	**78.72**	**63.00**	**1.11**	**0.74**	**15.00**	**17.50**	**1.78**	**1.73**	**49.34**	**54.70**	**1.75**	**1.77**
**Maximum**	**7.10**	**3.30**	**2.48**	**9.18**	**514.50**	**507.70**	**1.89**	**1.95**	**204.60**	**115.00**	**1.88**	**1.91**	**190.60**	**175.60**	**1.89**	**1.95**

Note: Data set in bold for Project A Ghana signifies purity that is beyond the acceptable range of 1.7–2.0. Data set in bold for Project B Nigeria signifies DNA shipped frozen on dry ice.

I-HAB, Institute of Human Virology Nigeria H3Africa Biorepository.

† , I-HAB compared its DNA concentration and purity results for the first pilot and first Phase II shipment with Project result’s.

Acceptable range for purity is 1.7–2.0, according to H3Africa’s submission guidelines.

The Phase II results displayed are a subset of 402 actual results; however, the averages and minimum and maximum values represent the 402 actual results.

‡ , I-HAB compared DNA concentration and purity results for the first pilot shipment conducted at controlled ambient and frozen temperatures, and the first Phase II shipment with results of Project B.

#### Continuous quality improvement

Through remediation, problems in DNA concentration and purity, and temperature monitoring improved as observed from Project A’s hub’s subsequent shipment to I-HAB. The first Phase II shipment occurred in April 2017. The shipment contained 402 DNA samples, 100% met H3Africa recommendations for concentration (Table 2). Purity improved to 38% acceptability per Ghana’s results and 45% per I-HAB. Temperature monitors confirmed that cold chain was maintained.

Phase II is ongoing. Thus, I-HAB continues to monitor compliance with shipping regulations and H3Africa requirements, and I-HAB will partner with Project A to devise resolutions if challenges arise. Through July 2019, Project A has deposited 15 333 non-DNA biospecimens at I-HAB for temporary storage and shipment to Ghana and deposited 7535 DNA samples for long-term storage.

### Project B (Nigeria)

#### Needs assessment

**FIGURE 3:**
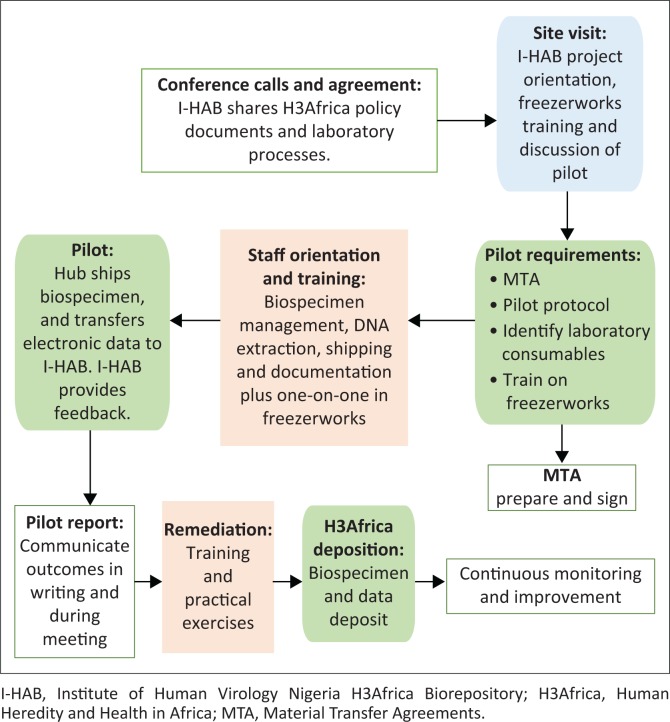
Partnership to empower Project B’s clinical site in Nigeria. I-HAB engaged clinical sites through needs assessment, training and mentorship, pilot exercise, and continuous monitoring in preparation for sample deposition.

Project B staff and I-HAB teams met three times: May, July and August 2014. The meetings facilitated an understanding of each other’s goals, requirements, expectations, and services. Consequently, Project B requested that I-HAB introduce biospecimens management during its staff orientation (Figure 3). The I-HAB manager and supervisor reviewed Project B’s study protocol to determine appropriate training topics.

#### Training and mentorship

The I-HAB supervisor attended Project B’s five-day study-wide orientation in July 2014, which included 35 research staff. The supervisor facilitated a biospecimen management workshop for one day, which spanned 14 topics (Table 3). She also reviewed supplies required for biospecimen collection, processing, and storage, and made recommendations, such as for laboratory supplies. For example, I-HAB suggested supplies for shipment of biological samples, Freezerworks basic LIMS software for sample management, and software for remote temperature monitoring and alerts, which remain in use by Project B. The I-HAB supervisor trained the project analyst in Freezerworks customisation and operations over five days, in late July 2014. Consequently, the analyst successfully customised the software, and adopted Freezerworks as the LIMS for biospecimen management.

**TABLE 3:** Training topics to build knowledge & skills – Project B in Nigeria†.

Training	Attendee type (No.)	Total number of staff trained	Venue	Topics covered
Project activation workshop (July 2014)	Medical doctor (20) Laboratory scientist (6) Research assistant (6) Community officer (5) Other professional (9)	35	Project B	Biospecimen collection DNA extraction: Theory and SOP only (no practical) Temperature monitoring Biospecimen shipment Biospecimen processing Documents, records, and data management Biospecimen storage Quality assurance
Freezerworks training (July 2014)	Programme analyst (1)	1	I-HAB	Freezerworks configuration User defined fields Entry form Barcode labels Import Export Freezers Security Freezerworks sample management Biospecimen entry Batch entry Batch update Simple and advanced searches Printing barcode labels
Pilot remediation (July 2015)	Laboratory scientist (1) Data officer (1)	2	Project B	Shipping documentation Biological shipping DNA QC DNA extraction Freezerworks navigation Standard preanalytical code H3A documentation

I-HAB, Institute of Human Virology Nigeria H3Africa Biorepository; SOP, standard operating procedures; H3A, Human Heredity and Health in Africa.

† , I-HAB provided three major trainings for Project B. The didactic training included theory and practical exercises.

#### Pilot

In January 2015 I-HAB drafted a protocol outlining pilot procedures. The teams attained ethical, legal, and regulatory requirements for the pilot. The MTA took one month to execute. In June 2015 Project B extracted, QC tested, and shipped biospecimens to I-HAB by road via TRANEX (Lagos, Nigeria) in one day. There were two shipments: one on dry ice (DNA-5, Plasma-4, and serum-5) and one at a controlled ambient temperature (DNA-5). I-HAB conducted DNA QC as specified above. Outcomes and recommendations were communicated.

The pilot identified areas that required improvement before Phase II. The hub forgot to send the manifest and the query form prior to the shipment. Both shipments contained insufficient refrigerant and were warm. The qualitative temperature indicators were not activated prior to shipment and the quantitative temperature loggers were misplaced. The logger for controlled ambient temperature was placed in the frozen shipment, while the logger for frozen shipments was placed in the controlled ambient temperature shipment. Nevertheless, 100% of the DNA had acceptable purity and concentration (Table 2). The benefit of the pilot was to learn of issues with biospecimen shipping procedures.

#### Continuous quality improvement

I-HAB provided remedial training for the laboratory manager and study coordinator in July 2015 (Table 3). The training occurred over five days and included biological packaging and shipping, operations of temperature indicators and monitors, DNA extraction, and DNA QC using NanoDrop. I-HAB demonstrated proper document completion over Skype calls. Following these preventative measures, the first Phase II shipment, containing 1000 DNA specimens, commenced without errors in documentation or packaging (November 2016). Temperature monitors demonstrated that the target temperature was maintained. Also, the concentration of DNA improved from 15.00 ng/*ml* – 69.66 ng/*ml* to 78.72 ng/*m* – 514 ng/*ml* and 100% of DNA was within acceptable range (Table 2).

Project B deposited 6000 DNA specimens as of September 2019. For each shipment I-HAB monitored the documentation, packaging, temperature monitors, and DNA QC. In May 2017, I-HAB noticed a decrease in DNA quality. Investigation revealed that a new employee had extracted the DNA; thus, I-HAB trained him in August 2018. DNA quality improved in the next shipment received in September 2018 and has remained within the recommended concentration and purity. I-HAB will continue to monitor related activities and liaise with Project B to investigate and resolve identified problems.

## Discussion

Researchers inexperienced with processes that influence biospecimen integrity may attain high-quality biospecimens for research by partnering with a biorepository and establishing a system of needs assessment, training and mentorship, pilot, and continuous quality improvement that is based on best practices. It is beneficial to start with general meetings and needs assessments to understand the intended goals and procedures and whether resources and capabilities are well aligned. Even if a scientist or contractor claims to be to be proficient, they should be subjected to a standardised assessment to ensure competency. Had Project A subjected its contractor to assessment, it would have learned of the contractor’s poor performance prior to study activation. The contractor had not resolved issues with purity that were indicative of protein contamination (purity ratio below 1.7) as of the first Phase I shipment. Likewise, Project B’s new hire experienced issues with DNA quality that could be circumvented via an assessment. Both examples demonstrate that assumptions of competency risk financial and biological resources. They also demonstrate the imperativeness of training and monitoring.

The key to effective training is to establish a foundation of harmonised guidelines, procedures, and minimal requirements that align with project goals and best practices. The biorepository should utilise standard and customised training that encompasses theory, practical exercises, and post-tests. Training achieved staff competency and eliminated issues revealed by needs assessments, pilot exercises, and continuous monitoring. Both projects improved in performance following training; however, biological shipping may require refresher training or additional practical exercises.

Training and mentorship contribute to the future cadre of knowledgeable researchers and generate business towards sustainability. Non-laboratorians demonstrated knowledge of biospecimen management after training. Freezerworks training empowered Project B’s analyst to customise and operate the LIMS. Also, the knowledge gained by Project A’s technologist empowered her to recommend changes to the study protocol, suggest areas of training for all sites, and train laboratory staff on Project A’s other clinical research sites. Project A and Project B requested additional services such as additional training and storage of non-DNA biospecimens. Both projects also gave public testimonials at scientific meetings that prompted interest and trust of other researchers.

The pilot process allows biorepositories to identify challenges in the cascade of processes leading to biospecimen deposit that may only be revealed by a real-time trial that involves all direct and indirect processes. For example, the pilots identified and eliminated errors in shipping that can only be unmasked by full trial. Due to the peculiarities of packaging and International Air Transport Association regulations associated with international biological shipments, on-site mentorship is useful to reinforce procedures learned during training, according to the trainees’ true environment and resources. Consequently, I-HAB incorporated site visits to assist sites to execute their first shipment to I-HAB.

Our outcomes mirrored the successes and challenges observed in other laboratory development programmes in sub-Saharan Africa that incorporate assessments, training, and mentorship. Generally, training and mentorship improved laboratory performance and empowered a new cadre of professionals.^14,15^ Problems in biospecimen acceptability,^3^ documents and records, information management, and supplies were common; however, training and mentorship facilitated improvements.^16,17^ Literature suggests that relapses may occur after training, particularly in documentation.^14^ Thus, I-HAB’s method of continuous improvement shall be critical to maintain improvements.

### Conclusion

Biobanking in Africa is expected to increase and improve with greater knowledge and resources.^6^ However, CRSs with abilities to meet best practices to collect, process, and store biospecimens of good integrity and quality are scarce. Partnerships between competent regional biorepositories and research investigators may build human capacity and improve biobanking practices and biospecimen quality by adopting an approach of needs assessment, training and mentorship, pilot, and continuous quality improvement. The improvements in quality may improve performance, increase staff morale, and contribute to sustainability of the biobank, research entity, and biobanking industry.

Lessons learned

- Clinical research sites and biorepositories may partner to empower clinical research sites to generate high-quality biospecimens via needs assessment, training, pilot, and continuous quality improvement.
- It is critical to standardise guidelines, procedures, and minimal criteria early on to inform training and activities.
- Standardised guidelines, procedures, and minimal criteria should be rooted in well-established requirements such as ISBER best practices, ISO 15189, and SLMTA.
